# Correspondence

**Published:** 1869-04

**Authors:** 


### Correspondence.

NASHVILLE, March 26, 1869.

PROF. TAFT—*Dear Sir:* I enclose you a communication from my friend, Professor Stout, hoping you will give it a place in the REGISTER. It shows an appreciation of our profession by Southern medical men that is gratifying. Humanity demands that Dentists should be assigned to duty in all armies and army hospitals. Their attainments in the healing art will justify it. It will be seen from this paper that the South takes the initiative (so far as I know) in this matter, as she has done in the organization of Dental Colleges and the enactment of State laws for the protection of our profession, and in the interests of humanity, in this direction.

I hope that other governments (if not all governments in the civilized world) will move toward making such appointments, and thus be found following in the footsteps of the late "so called" Confederate authorities in this particular.

Very truly, W. H. MORGAN.

---

NASHVILLE, TENN., January 11, 1868.

W. H. MORGAN, M. D., D. D. S.—*Dear Sir:* Agreeably to your request, I commit the following reminiscences to writing:

During the year 1863, while Medical Director of the General Hospitals of the Confederate Army of Tennessee, I was called upon in the City of Atlanta, by an officer, to administer chloroform to his wife, who expected to have a number of teeth drawn by Dr. Bean, D. D. S., then pracicing Dentistry on Marietta street. Dr. B., then a stranger, excited my admiration by the dexterity and skill with which he extracted the lady's teeth, upon which I sincerely complimented him. This drew us into a protracted conversation in regard to Dentistry and Surgery in general. Dr. B. tendered his services at least one day in every week to perform operations upon the teeth of soldiers in hospitals in Atlanta, which I gladly accepted, as I found him highly intelligent and benevolently disposed. I directed the Post Surgeon, Dr. J. P. Logan, now Professor of Theory and Practice in Washington University, Baltimore, to inform the surgeons in charge of hospitals in the city of the tender made by Dr. B. Soon Dr. B.'s visits to the hospital suggested to Dr. W. F. Westmoreland, now Prof. of Surgery in the Atlanta Medical College, then Surgeon in charge of the Medical College Hospital, that Dr. B.'s skill in Mechanical Dentistry might be made use of in the treatment of gun shot fracture of the inferior maxilla.

The result of the suggestions of Dr. W. and the ingenuity of Dr. B. was the production of a splint, which might be said to have been a perfect success. This splint was described by Dr. E. N. Covey, in an early number of the first volume of the *Richmond Medical Journal*, to which I refer you. So successful was this splint in the treatment of this class of cases, that I ordered the setting apart of a ward in the Medical College Hospital, to be devoted to the treatment of fractures of the lower jaw, and thither ordered all the cases in the department for treatment. When Atlanta was evacuated, I transferred the patients in this ward to the Blind Asylum Hospital, in Macon, whither Dr. Bean was also sent to continue the services begun in Atlanta. Reports of the success of the treatment were sent to Surgeon General S. P. Moore, who ordered Dr. Bean in person to Richmond, where the value of the mechanical appliance he had invented was acknowledged.

Early in the history of my Medical Directorship, I encouraged the detail of skilled Dentists for service in the hospitals, and before the termination of the war almost every hospital of large size had one Dentist in it, doing service upon no more than detailed soldiers pay. The value of their services had so impressed the Surgeon General, that about two months before the surrender of the Confederate armies, he sent out blank forms of returns of Dental operations performed. I do not remember that any of these were ever returned. Should I ever be able to lay my hands upon one of those blanks, I will hand it to you as worthy of preservation as a memento of the acknowledgement of the importance of Dental Surgery by the highest officer of the Medical Bureau of the Confederate States.

The Surgeon General could find no authority of law to grant higher compensation than that of hospital steward to those detailed as Dentists in the hospitals. It is with pleasure that I bear testimony to the alacrity with which Surgeon Dentists co-operated with military surgeons in the Southern service, and to the skill which many of them displayed with the meager means and materials at their command. I doubt not that had the war continued a year longer, provision would have been made for the recognition by law of the office of Dentist in the Confederate service.

If the above facts, which may be implicitly relied upon, will be of any service in inspirating the members of your profession to the maintenance of a high standard of professional education and excellence, you are at liberty to make use of them as you may see fit. Very respectfully yours,

S. H. Stout, M. D.

